# 

**Published:** 1884-12

**Authors:** 


ORIGINAL COMMUNICATIONS:

Professional Services and Professional Fees. E. T. Darby ............ 653
Personal Recollections of a Dentist of the Early Days. L. W. Bristol. 662
Suggestions for the Construction of Artificial Crowns and Bridges. J. H. Spaulding ............ 670
Senile Decay. J. Smith Dodge, Jr ............ 675
The Transplanting and Replanting of Teeth. A. H. Fuller ............ 677
An Objectionable Clause. W. C. Starbuck ............ 678

REPORTS OF SOCIETY MEETINGS:

American Medical Association ............ 679
Pennsylvania State Dental Society ............ 683
American Dental Society of Europe ............ 689
American Dental Association ............ 693
Central Dental Association of Northern New Jersey ............ 699

EDITORIAL:

Dead Teeth in the Jaws ............ 702
A Model Report ............ 707

CURRENT NEWS AND OPINION:

Legal Decisions ............ 661
Progress under Difficulties ............ 682
Dental School in Berlin ............ 707
Obtuse ............ 708
Muriate of Cocoaine ............ 708
The Ohio State Dental Society ............ 708
Askings and Answers ............ 709

